# A phase I/II study of rovalpituzumab tesirine in delta-like 3—expressing advanced solid tumors

**DOI:** 10.1038/s41698-021-00214-y

**Published:** 2021-08-05

**Authors:** Aaron S. Mansfield, David S. Hong, Christine L. Hann, Anna F. Farago, Himisha Beltran, Saiama N. Waqar, Andrew E. Hendifar, Lowell B. Anthony, Matthew H. Taylor, Alan H. Bryce, Scott T. Tagawa, Karl Lewis, Jiaxin Niu, Christine H. Chung, James M. Cleary, Michael Rossi, Carrianne Ludwig, Ricardo Valenzuela, Yan Luo, Rahul Aggarwal

**Affiliations:** 1 Mayo Clinic, Rochester, MN USA; 2 The University of Texas MD Anderson Cancer Center, Houston, TX USA; 3 Johns Hopkins Sidney Kimmel Comprehensive Cancer Center, Baltimore, MD USA; 4 Massachusetts General Hospital, Boston, MA USA; 5 Dana-Farber Cancer Institute, Boston, MA USA; 6 Washington University School of Medicine, St. Louis, MO USA; 7 Cedars-Sinai Medical Center, Los Angeles, CA USA; 8 University of Kentucky Chandler Medical Center, Lexington, KY USA; 9 Earle A. Chiles Research Institute, Providence Cancer Institute, Portland, OR USA; 10 Mayo Clinic, Phoenix, AZ USA; 11 Weill Cornell Medicine, New York, NY USA; 12 University of Colorado Denver, Aurora, CO USA; 13 Banner MD Anderson Cancer Center, Gilbert, AZ USA; 14 H Lee Moffitt Cancer Center, Tampa, FL USA; 15 AbbVie, Inc, North Chicago, IL USA; 16 UCSF Helen Diller Family Comprehensive Cancer Center, San Francisco, CA USA

**Keywords:** Phase II trials, Targeted therapies, Phase I trials

## Abstract

Delta-like protein 3 (DLL3) is highly expressed in solid tumors, including neuroendocrine carcinomas/neuroendocrine tumors (NEC/NET). Rovalpituzumab tesirine (Rova-T) is a DLL3-targeting antibody-drug conjugate. Patients with NECs and other advanced DLL3-expressing tumors were enrolled in this phase I/II study (NCT02709889). The primary endpoint was safety. Two hundred patients were enrolled: 101 with NEC/NET (large-cell NEC, gastroenteropancreatic NEC, neuroendocrine prostate cancer, and other NEC/NET) and 99 with other solid tumors (melanoma, medullary thyroid cancer [MTC], glioblastoma, and other). The recommended phase II dose (RP2D) was 0.3 mg/kg every 6 weeks (q6w) for two cycles. At the RP2D, grade 3/4 adverse events included anemia (17%), thrombocytopenia (15%), and elevated aspartate aminotransferase (8%). Responses were confirmed in 15/145 patients (10%) treated at 0.3 mg/kg, including 9/69 patients (13%) with NEC/NET. Rova-T at 0.3 mg/kg q6w had manageable toxicity, with antitumor activity observed in patients with NEC/NET, melanoma, MTC, and glioblastoma.

## Introduction

Delta-like protein 3 (DLL3) is a ligand in the Notch signaling pathway that is highly expressed in tumors of neuroendocrine origin but not in normal tissues^1,2^. The Notch signaling pathway regulates cell proliferation, differentiation, and cell death and may have tumor-suppressive or oncogenic effects, depending on the tissue microenvironment^3^. Suppression of the *NOTCH* gene has been shown to promote oncogenesis in small cell lung cancer (SCLC), medullary thyroid carcinoma (MTC), and pancreatic and biliary neuroendocrine tumors^3,4^. Although the function of DLL3 is not fully understood, it has been implicated in the inhibition of the Notch signaling pathway in the regulation of cell development and cell fate decisions^3,5^.

Neuroendocrine carcinomas (NEC) are a group of poorly differentiated neuroendocrine neoplasms^3^ that commonly express DLL3, with positive DLL3 expression observed in 65–74% of large cell NEC and in 77% of castration-resistant neuroendocrine prostate cancer (NEPC)^6,7^. In contrast, DLL3 expression is not observed at a high prevalence in low-grade, well-differentiated neuroendocrine tumors (NET). Due to the heterogeneity and rarity of neuroendocrine neoplasms, they are understudied and poorly understood^3^. Platinum-based chemotherapy is a standard first-line option for NEC, despite the lack of survival advantage demonstrated in randomized trial^8–10^. Overall survival (OS) in patients with NEC is <18 months^9–13^. A significant need beyond first-line therapy exists for novel therapeutic treatment options for patients with NEC, and DLL3 is a potential therapeutic target.

In addition to NEC, other cancers have high DLL3 expression, including melanoma, MTC, and glioblastoma (GBM)^14^. Patients with metastatic melanoma are typically treated with immune checkpoint inhibitors and BRAF and MEK inhibitors, which produce high response rates with impressive durability. However, metastatic melanoma will ultimately become refractory to these therapies, and median progression-free survival (PFS) is <1 year with these agents^15,16^. MTC makes up 1–2% of thyroid cancers, and 10–15% of patients present with metastatic disease at diagnosis. These patients are typically treated with a multikinase inhibitor, such as cabozantinib or vandetanib, or with selpercatinib or other RET inhibitors for those with RET-mutated tumors^17^. However, new treatment options are needed for patients with MTC who do not benefit from or are intolerant to these agents. GBM accounts for 54% of all gliomas, and initial treatment often consists of surgery, radiation therapy, and systemic therapy^18,19^. Only one-third of patients will survive for 1 year, and <5% of patients will live beyond 5 years^18^. Given the response to currently available treatments, novel approaches are needed to treat patients with these types of cancers.

Rovalpituzumab tesirine (Rova-T) is a first-in-class antibody-drug conjugate that targets DLL3 to deliver a cytotoxic compound directly to tumor cells. Rova-T is composed of a monoclonal DLL3 antibody linked to a DNA intercalating agent (pyrrolobenzodiazepine) via a protease-cleavable linker. In SCLC and large cell neuroendocrine patient-derived xenograft models, Rova-T significantly inhibited tumor growth compared with standard platinum-based therapy by effectively targeting and eliminating DLL3-positive tumor-initiating cells^2^. A phase I study (NCT01901653) demonstrated Rova-T antitumor activity in patients with recurrent SCLC^20^. The objective response rate (ORR) was 31%, and the 1-year survival rate was 32%; the median OS was 5.8 months in patients with tumors expressing a high level of DLL3. In the phase II TRINITY study (NCT02674568) of Rova-T in patients with relapsed/refractory SCLC, the ORR was 14%, the median PFS was 3.8 months, and the median OS was 5.7 months in patients with tumors expressing a high-level DLL3^21^.

Given the activity of Rova-T observed in studies of SCLC and the prevalence of DLL3 expression in solid tumors described above, this study examined the safety, tolerability, and antitumor activity of Rova-T in patients with DLL3-positive tumors, including NEC/NET, melanoma, MTC, GBM, and other solid tumors.

## Results

### Patient demographics, baseline characteristics, and disposition

Between September 2016 and February 2019, 1293 patients were pre-screened for DLL3-positive tumor status (Supplementary Table 1); ~287 patients were ultimately screened for the study, and 200 patients were subsequently enrolled and received at least one dose of Rova-T. Disease-specific cohorts included both a dose-escalation and expansion cohort. As of the data cutoff of October 14, 2019, patients had received a median of one cycle of therapy (range, 1‒5); 96 patients (48%) had received two or more cycles of Rova-T.

There were 101 patients with NEC/NET (pulmonary and extrapulmonary large cell NEC [*n* = 13], NEPC [*n* = 21], high-grade gastroenteropancreatic [GEP] NEC [*n* = 36], and other NEC/NET [*n* = 31]) and 99 patients with other solid tumors (melanoma [*n* = 20], MTC [*n* = 13], GBM [*n* = 23], and other [*n* = 43]). Supplementary Table 2 provides a breakdown of primary diagnosis for other NEC/NET. The median age was 61 (range, 28‒84) years, and 94% of patients had stage IV disease at study entry (Table 1). Seventy-seven (39%) patients had tumors expressing a high level of DLL3, which was defined as ≥50% DLL3-positive cells. Most patients (55%) had received three or more prior therapies. The median duration of follow-up was 4.6 (range, 0.1–33.7) months in all patients and 4.7 (range 0.1–27.1) months in patients treated at the recommended phase 2 dose (RP2D) of 0.3 mg/kg. The experience of one patient was described previously^22^.

**Table 1:** Patient demographics and baseline characteristics.

Characteristic	Melanoma (*n* = 20)	MTC (*n* = 13)	GBM (*n* = 23)	Other solid tumor (*n* = 43)	Pooled NEC/NET (*n* = 101)	All treated patients (*N* = 200)
Median age (range), years	63.5 (40–78)	60 (33–67)	57 (36–72)	64 (28–84)	61 (28–82)	61 (28–84)
Male sex, *n* (%)	10 (50)	8 (62)	19 (83)	23 (53)	66 (65)	126 (63)
ECOG performance status, *n* (%)						
0	7 (35)	1 (8)	1 (4)	5 (12)	20 (20)	34 (17)
1	13 (65)	12 (92)	22 (96)	38 (88)	80 (79)	165 (83)
2	0	0	0	0	1 (1)	1 (1)
Disease stage at study entry						
Stage IIa	1 (5)	0	0	0	0	1 (1)
Stage IIb	0	0	0	2 (5)	0	2 (1)
Stage IIIa	0	0	0	1 (2)	3 (3)	4 (2)
Stage IIIb	0	0	0	0	2 (2)	2 (1)
Stage IIIc	1 (5)	0	0	1 (2)	0	2 (1)
Stage IV	18 (90)	12 (92)	23 (100)	39 (91)	95 (94)	187 (94)
Missing	0	1 (8)	0	0	1 (1)	2 (1)
DLL3 level, *n* (%)						
High (≥50%)	5 (25)	6 (46)	2 (9)	11 (26)	53 (52)	77 (39)
Low (1–49%)	14 (70)	7 (54)	21 (91)	32 (74)	48 (48)	122 (61)
0%	1 (5)^a^	0	0	0	0	1 (0.5)
Prior lines of therapy, n (%)						
1	3 (15)	4 (31)	2 (9)	6 (14)	22 (22)	37 (19)
2	4 (20)	4 (31)	7 (30)	10 (23)	25 (25)	50 (25)
3	5 (25)	0	6 (26)	8 (19)	27 (27)	46 (23)
≥4	8 (40)	2 (15)	8 (35)	19 (44)	27 (27)	64 (32)
Missing	0	3 (23)	0	0	0	3 (2)
Response to first-line therapy, *n* (%)^b^						
Sensitive^c^	4 (20)	1 (8)	4 (17)	15 (35)	33 (33)	57 (29)
Resistant^d^	3 (15)	4 (31)	4 (17)	3 (7)	17 (17)	31 (16)
Refractory^e^	7 (35)	2 (15)	5 (22)	12 (28)	19 (19)	45 (23)
Undetermined	6 (30)	6 (46)	10 (43)	13 (30)	32 (32)	67 (34)

^a^The patient had two DLL3 results; the result with a value of 0 was the latest value before enrollment and therefore was used in the analysis.

^b^Refers to platinum-based therapy only.

^c^Sensitive is defined as a first-line response that is neither refractory nor undetermined, and the start date of second-line treatment is ≥90 days after the end of the first-line treatment^31^.

^d^Resistant is defined as a first-line response that is neither refractory nor undetermined, and the start date of second-line treatment is <90 days after the end of the first-line treatment^31^.

^e^Refractory if a first-line response is a progressive disease.

Rova-T was administered at dose levels of 0.2 mg/kg (*n* = 43), 0.3 mg/kg (*n* = 145), and 0.4 mg/kg (*n* = 12). Reasons for discontinuation of Rova-T included disease progression (*n* *=* 58, 29%), adverse events (AEs) (*n* *=* 48, 24%), investigator decision (*n* *=* 21, 11%), withdrawn consent (*n* *=* 24, 12%), and other reasons (treatment completed: *n* *=* 14, 7%; clinical progression: *n* *=* 10, 5%; withdrawn for hospice or other treatment: *n* *=* 3, 2%; lost to follow-up: *n* *=* 2, 1%; noncompliance: *n* *=* 2, 1%; death: *n* *=* 1, 1%; and unknown reasons: *n* *=* 17, 9%).

### Dose escalation findings: dose-limiting toxicities (DLTs) and RP2D

Overall, seven DLTs were experienced by five patients in this study. At the 0.2-mg/kg dose level, two of 43 (5%) patients experienced DLTs, including one patient with grade 3 photosensitivity reaction and one patient with grade 3 dyspnea. Two of 145 (3%) patients in the 0.3-mg/kg dose level had DLTs, including one patient with grade 2 effusion and one patient with grade 3 rhabdomyolysis, grade 3 tumor lysis syndrome, and grade 4 kidney injury. One of 12 (8%) patients treated at the 0.4-mg/kg dose level had a DLT of grade 4 thrombocytopenia. Despite only one DLT identified in the 0.4-mg/kg group, the safety data in totality indicated that 0.4 mg/kg every six weeks (q6w) is not well tolerated (Table 2). Of the 12 patients enrolled in that cohort, nine of 12 (75%) patients had grade 3/4 AEs, and six experienced drug-related serious AEs (SAEs), including one patient with grade 5 hepatic failure. Because of these safety findings, 0.3 mg/kg q6w for two cycles was chosen as the RP2D.

**Table 2:** Summary of overall safety by dose.

AE, *n* (%)	0.2 mg/kg (*n* = 43)	0.3 mg/kg (*n* = 145)	0.4 mg/kg (*n* = 12)	All (*N* = 200)
Any TEAE	42 (98)	144 (99)	12 (100)	198 (99)
Grade 3/4 TEAE	30 (70)	78 (54)	9 (75)	117 (59)
Drug-related TEAE	37 (86)	132 (91)	12 (100)	181 (91)
Drug-related grade 3/4 TEAE	20 (47)	70 (48)	7 (58)	97 (49)
SAE	26 (60)	77 (53)	8 (67)	111 (56)
Drug-related SAEs	9 (21)	34 (23)	6 (50)	49 (25)
TEAE leading to death	4 (9)	21 (14)	2 (17)	27 (14)
Drug-related TEAE leading to death	0	4 (3)	2 (17)	6 (3)

### Clinical safety

Most patients (144 of 145; 99%) treated with 0.3 mg/kg Rova-T had at least one treatment-emergent AE (TEAE) (Table 3). The most common all-grade TEAEs were fatigue in 75 patients (52%), nausea in 53 patients (37%), and thrombocytopenia and pleural effusion in 48 patients (33%) each. Grade 3/4 AEs occurred in 78 of 145 (54%) patients. Fifty-nine (42%) patients had SAEs (excluding malignant neoplasm progression), most commonly pleural effusion (*n* = 7; 5%), pericardial effusion (*n* = 6; 4%), and dyspnea (*n* = 5; 3%; Supplementary Table 3). TEAEs of special interest were pleural effusion (*n* = 48; 33%), peripheral edema (*n* = 44; 30%), pericardial effusion (*n* = 38; 26%), photosensitivity reaction (*n* = 37; 26%), and pneumonitis (*n* = 3; 2%). For each of these, grade 3/4 events occurred in <5% of the overall population (Supplementary Table 4). Overall, 31 (21%) patients discontinued treatment due to TEAEs.

**Table 3:** TEAEs in >15% at any grade in all patients treated at the 0.3-mg/kg dose.

Preferred term, *n* (%)	Melanoma (*n* = 17)		MTC (*n* = 10)		GBM (*n* = 18)		Other solid tumor (*n* = 31)		Pooled NEC/NET (*n* = 69)		All patients treated at 0.3 mg/kg (*N* = 145)	
	All	Grade 3/4	All	Grade 3/4	All	Grade 3/4	All	Grade 3/4	All	Grade 3/4	All	Grade 3/4
Any TEAE	16 (94)	8 (47)	10 (100)	6 (60)	18 (100)	8 (44)	31 (100)	16 (52)	69 (100)	40 (58)	144 (99)	78 (54)
Fatigue	9 (53)	1 (6)	4 (40)	0	10 (56)	1 (6)	13 (42)	2 (6)	39 (57)	2 (3)	75 (52)	6 (4)
Nausea	5 (29)	0	3 (30)	0	4 (22)	1 (6)	12 (39)	2 (6)	29 (42)	2 (3)	53 (37)	5 (3)
Thrombocytopenia	2 (12)	0	2 (20)	1 (10)	3 (17)	1 (6)	11 (35)	4 (13)	30 (43)	16 (23)	48 (33)	22 (15)
Pleural effusion	6 (35)	0	3 (30)	0	5 (28)	0	7 (23)	2 (6)	27 (39)	2 (3)	48 (33)	4 (3)
Peripheral edema	5 (29)	0	3 (30)	0	3 (17)	0	9 (29)	0	24 (35)	1 (1)	44 (30)	1 (1)
Decreased appetite	6 (35)	0	0	0	3 (17)	1 (6)	11 (35)	1 (3)	22 (32)	1 (1)	42 (29)	3 (2)
Anemia	3 (18)	0	1 (10)	1 (10)	1 (6)	1 (6)	14 (45)	9 (29)	24 (35)	13 (19)	43 (30)	24 (17)
Dyspnea	5 (29)	1 (6)	5 (50)	2 (20)	3 (17)	0	6 (19)	1 (3)	21 (30)	0	40 (28)	4 (3)
Pericardial effusion	5 (29)	0	4 (40)	1 (10)	5 (28)	1 (6)	5 (16)	1 (3)	19 (28)	2 (3)	38 (26)	5 (3)
Photosensitivity reaction	5 (29)	0	5 (50)	0	5 (28)	0	6 (19)	0	16 (23)	3 (4)	37 (26)	3 (2)
Vomiting	3 (18)	0	1 (10)	0	6 (33)	1 (6)	8 (26)	2 (6)	15 (22)	2 (3)	33 (23)	5 (3)
Abdominal pain	2 (12)	0	2 (20)	1 (10)	2 (11)	0	6 (19)	1 (3)	18 (26)	3 (4)	30 (21)	5 (3)
Aspartate aminotransferase elevation	0	0	2 (20)	2 (20)	3 (17)	0	5 (16)	2 (6)	19 (28)	8 (12)	29 (20)	12 (8)
Constipation	2 (12)	0	2 (20)	0	1 (6)	0	5 (16)	0	19 (28)	1 (1)	29 (20)	1 (1)
Alanine aminotransferase elevation	0	0	3 (30)	1 (10)	3 (17)	1 (6)	3 (10)	0	17 (25)	6 (9)	26 (18)	8 (6)
Cough	3 (18)	0	2 (20)	0	2 (11)	0	4 (13)	0	14 (20)	0	25 (17)	0
Diarrhea	3 (18)	0	0	0	2 (11)	0	4 (13)	1 (3)	14 (20)	2 (3)	23 (16)	3 (2)

At the time of data cutoff, a total of 21 of 145 (14%) patients who were treated at the 0.3-mg/kg dose level experienced a grade 5 TEAE; 14 of 145 (10%) patients had a grade 5 event of malignant neoplasm progression, and one of 145 (<1%) patients had a grade 5 event of another malignancy. Six of 145 (4%) patients experienced a TEAE leading to death that was not related to disease progression or malignancy; grade 5 TEAEs that were not related to disease progression or malignancy included two events of pneumonitis, and one event each of multiple organ dysfunction, acute respiratory failure, hepatic encephalopathy, device-related infection, and acute kidney injury (Supplementary Table 5). Four of 145 (3%) patients had AEs leading to death that were related to Rova-T, including two who died of pneumonitis, one who died of acute respiratory failure, and one who died of hepatic encephalopathy.

### Efficacy

One hundred forty-five patients received at least one dose of Rova-T at 0.3 mg/kg and were included in the efficacy analyses (Table 4). The median follow-up for patients treated at 0.3 mg/kg was 4.7 months (range, 0.1–27.1). Overall, the ORR was 10%, including one complete response (CR) and 14 partial responses (PRs), and the best overall response (BOR) rate was 17% (25/145; one CR and 24 PRs). In pooled patients with NEC/NET, the ORR was 13% (9/69; all PRs) and the BOR rate was 25% (17/69; all PRs) (Supplementary Table 6). The median PFS was 4.1 months (95% CI, 2.8–4.8), and the median OS was 7.1 months (95% CI, 5.6–9.7) in pooled patients with NEC/NET. Efficacy for 43 patients treated with Rova-T at 0.2 mg/kg and 12 patients treated at 0.4 mg/kg is reported in Supplementary Tables 7 and 8. The best change in tumor lesion size in patients in each cohort is shown in Supplementary Figs. 1–8.

**Table 4:** Efficacy at the 0.3-mg/kg dose.

Outcome	Melanoma (*n* = 17)	MTC (*n* = 10)	GBM (*n* = 18)	Other solid tumor (*n* = 31)	Pooled NEC/NET (*n* = 69)
ORR, *n* (%) (95% CI)	1 (5.9) (0.1–28.7)	2 (20.0) (2.5–55.6)	1 (5.6) (0.1–27.3)	2 (6.5) (0.8–21.4)	9 (13) (6.1–23.3)
CR	0	0	1 (5.6)	0	0
PR	1 (5.9)	2 (20.0)	0	2 (6.5)^a^	9 (13)
BOR, *n* (%)	2 (11.8)	2 (20.0)	1 (5.6)	3 (9.7)	17 (24.6)
CR	0	0	1 (5.6)	0	0
PR	2 (11.8)	2 (20.0)	0	3 (9.7)	17 (24.6)
Median DOR (95% CI), months^b^	2.9 (NE–NE)	NR (4.5–NE)	4.6 (NE–NE)	3.9 (0.4–4.1)	3.1 (2.3–NE)
Median PFS (95% CI), months	2.9 (1.3–3.7)	11.7 (1.3–NE)	1.4 (1.2–2.5)	1.8 (1.3–4.0)	4.1 (2.8–4.8)
Median OS (95% CI), months	6.4 (3.3–12.8)	NR (6.0–NE)	6.6 (2.8–9.7)	4.9 (3.9–5.9)	7.1 (5.6–9.7)

*NE* not estimable, *NR* not reached.

^a^Patients with confirmed PRs in the “Other solid tumor” category include 1 with neuroendocrine carcinoma of the lung and 1 with small cell carcinoma of the esophagus.

^b^DOR is defined as the time from the first assessment on therapy of a CR or PR to the date of disease progression.

In pooled patients with NEC/NET expressing a high level of DLL3 (≥50% DLL3-positive tumor cells), the ORR was 17% (6/35) and 34% (12/35) had a BOR (all PRs). In those with NEC/NET expressing a low level of DLL3 (1–49% DLL3-positive tumor cells), the ORR was 9% (3/34) and the BOR rate was 15% (5/34) (all PRs; Table 5). The median PFS values for pooled patients with NEC/NET expressing high and low levels of DLL3 were 4.3 months (95% CI, 2.7–6.1) and 3.3 months (95% CI, 2.4–4.8), respectively. The median OS values for patients expressing high and low levels of DLL3 were 7.4 months (95% CI, 5.6–13.1) and 7.1 months (95% CI, 4.3–9.9), respectively.

**Table 5:** Efficacy for DLL3-high and DLL3-low expression in Pooled NEC/NET at the 0.3-mg/kg dose.

Outcome	Pooled NEC/NET	
	DLL3 high (*n* = 35)	DLL3 low (*n* = 34)
ORR (95% CI), *n* (%)	6 (17.1) (6.6–33.6)	3 (8.8) (1.9–23.7)
CR	0	0
PR	6 (17.1)	3 (8.8)
BOR, *n* (%)	12 (34.3)	5 (14.7)
CR	0	0
PR	12 (34.3)	5 (14.7)
Median DOR (95% CI), months^a^	2.9 (1.2–NE)	NR (3.7–NE)
Median PFS (95% CI), months	4.3 (2.7–6.1)	3.3 (2.4–4.8)
Median OS (95% CI), months	7.4 (5.6–13.1)	7.1 (4.3–9.9)

NE not estimable.

^a^DOR is defined as the time from the first assessment on therapy of a CR or PR to the date of disease progression.

## Discussion

In this multicenter, open-label, phase I/II study, the safety and efficacy of Rova-T monotherapy were evaluated at three dose levels across advanced solid tumors with DLL3 expression. Patients treated at the 0.2-mg/kg and 0.3-mg/kg dose levels had fewer drug-related SAEs (21% and 23%, respectively) compared with those treated at 0.4 mg/kg (50%). Patients treated at the lower dose levels also had fewer drug-related TEAEs leading to death (0–3%) compared with those treated at 0.4 mg/kg (17%). Accordingly, the RP2D of Rova-T was chosen as 0.3 mg/kg q6w for two cycles with the option of treatment beyond two cycles or retreatment upon progression based on individual risk-benefit balance. The TEAEs reported in this study were similar to those observed previously with Rova-T in patients with relapsed/refractory SCLC^20,21^. High rates of pleural effusion, peripheral edema, pericardial effusion, and photosensitivity were observed in patients treated at 0.3 mg/kg. The rate of any-grade pericardial effusion in this study (26%) was higher than reported in previous studies of Rova-T (14–16%)^20,21^. Grade 3/4 TEAEs of special interest occurred at low rates. Overall, Rova-T at 0.3 mg/kg q6w had a more manageable toxicity profile than did 0.4 mg/kg for the majority of patients, and no appreciable differences in toxicity profiles were observed across the tumor types evaluated. Owing to the small sample sizes, it is not possible to conclude whether small differences in the toxicity profiles between different tumor types or between the 0.2 mg/kg and 0.3 mg/kg dose levels were statistically significant; however, the 0.4 mg/kg dose was clearly more toxic. Strategies to mitigate toxicity that were utilized in this study included premedication with steroids, educating investigators on important risks (pleural/pericardial effusions, edema, photosensitivity, and pneumonitis), reminding investigators to closely monitor for risks and manage with standard clinical practice, and sharing of best practices among investigators.

Toxicities associated with Rova-T treatment, including pleural and pericardial effusions, may be caused in part by the pyrrolobenzodiazepine component of the antibody-drug conjugate. While the mechanism is not fully understood, studies suggest that systemic release or bystander effect may be involved^22–24^. Systemic release occurs when premature cleavage of the linker results in the release of the drug into circulation, causing off-target toxicities. Bystander effect is the diffusion of the drug from the target cell to neighboring cells that do not have the target protein, either by leaking from the targeted cell or cleavage of the drug before it is internalized. In either case, DLL3-negative cells may be inappropriately exposed to pyrrolobenzodiazepine, and further refinement of the linker or the drug-antibody ratio may mitigate these effects.

This study enrolled a heavily pretreated population of patients with DLL3-expressing tumors, and 55% of patients received at least three prior lines of therapies. These findings demonstrate that Rova-T as a single agent had antitumor activity in a subset of heavily pretreated patients with tumor types that express DLL3, including NEC/NET, melanoma, and MTC. These tumor types tend to be treatment refractory following multiple prior lines of therapy, and this may have contributed to the low response rate. In addition, although DLL3 expression by IHC was required for study entry, intratumoral variability in DLL3 expression may have limited the overall antitumor activity of Rova-T. The median PFS and OS in the patients in this study with the refractory disease were similar to ranges shown in previous studies^25–28^.

Despite the limited sample size, a trend toward a higher response rate was observed among patients with NEC/NET with high DLL3 expression, supporting DLL3 as a promising therapeutic target for the treatment of NEC/NET across primary disease sites, including treatment-emergent NEPC. For pooled patients with NEC/NET, the duration of response (DOR) of 3.1 months appears comparable to the efficacy with common treatment for these tumor types in the relapsed or refractory setting^29^. These findings are of particular interest for patients with recurrent NEC, for which there is no standard-of-care treatment. Of note, the category of grade 3 NET was not established for pancreatic and gastrointestinal NET for most of the study enrollment, therefore it is not known whether any patients with grade 3 GEP NET were part of the study. The majority of patients who were enrolled in the other NEC/NET cohort were classified as having NEC, so it was not possible to determine whether there was a difference in DLL3 expression in NET versus NEC.

Overall, refinement of both the drug-antibody ratio and the linker in Rova-T could improve drug delivery, reduce toxicity, and increase treatment duration, which could lead to improved efficacy. Further study is needed to define the risk-benefit balance of Rova-T in patients with NEC/NET. During the conduct of the current trial, further development of Rova-T was discontinued based on results from two phase III studies that indicated a lack of favorable risk-benefit balance of Rova-T in patients with SCLC (https://news.abbvie.com/news/press-releases/abbvie-discontinues-rovalpituzumab-tesirine-rova-t-research-and-development-program.htm; https://news.abbvie.com/news/phase-3-trial-rova-t-as-second-line-therapy-for-advanced-small-cell-lung-cancer-tahoe-study-halted.htm). However, DLL3 remains a relevant anticancer target. Additional strategies targeting DLL3 in these difficult-to-treat tumor types warrant investigation.

## Methods

### Patients

Adult patients with unresectable, refractory, advanced solid tumors other than SCLC who were positive for DLL3 and had measurable disease were included in the study. DLL3 positivity was defined as immunohistochemical staining in ≥1% of tumor cells. Potential patients were pre-screened for DLL3 positivity to determine initial eligibility, and those with DLL3-positive tumors underwent full screening for study eligibility upon disease progression. Measurable disease was defined based on Response Evaluation Criteria in Solid Tumors version 1.1 (RECIST v1.1)^30^. Patients had to have an Eastern Cooperative Oncology Group (ECOG) performance status of 0 to 1, a life expectancy of ≥12 weeks, and satisfactory laboratory parameters. Patients could not have a clinically significant medical condition, including uncontrolled hypertension and/or diabetes, pulmonary disease, neurological disorder, recent or ongoing serious infection, or a cerebral vascular event within six months of starting the study. Prior exposure to pyrrolobenzodiazepine-containing drugs, including Rova-T, was not allowed. All patients provided written informed consent.

### Study design and objectives

This multicenter, open-label, phase I/II study (NCT02709889) enrolled patients in the United States. The study was conducted according to the Declaration of Helsinki and all applicable laws, rules, and regulations within the relevant jurisdictions of the investigators; the study was approved by the institutional review boards at each participating institution. Patients were enrolled in disease-specific cohorts, including melanoma, MTC, GBM, large-cell NEC, NEPC, GEP NEC, other NEC/NET, and other solid tumors. Dosing was predetermined to start at 0.2 mg/kg q6w, which is one dose level below the RP2D for SCLC, with dose escalation continuing through 0.4 mg/kg, the maximum tolerated dose for SCLC^20^. In Part A, the RP2D was determined in disease-specific cohorts. Part B tested the RP2D determined in Part A in disease-specific expansion cohorts. The primary endpoint was safety. Secondary endpoints included BOR, ORR, DOR, PFS, and OS. The relationship between DLL3 expression and clinical outcome was tested as an exploratory endpoint.

### Treatment and assessments

DLL3 expression was determined at baseline with fresh or archived tumor tissue using an SC16.65 mouse antibody IHC investigational use only (IUO) assay developed by Ventana Medical Systems as previously described, at a concentration of 0.78 µg/ml (AbbVie Stemcentrix, Lot No. 170420)^20,21^. Rova-T was administered intravenously on day 1 of each 6-week cycle at 0.2, 0.3, or 0.4 mg/kg with a 3 + 3 design for dose escalation in Part A. Patients received treatment until disease progression or unacceptable toxicity in Part A. Patients in Part B received two doses and could receive further doses at the discretion of the investigator. Dosing interval and duration were selected based on previous clinical studies of Rova-T in SCLC^20,21^. Dexamethasone (8 mg) was administered orally twice daily on the day before treatment with Rova-T and on days 1 and 2 of treatment in each cycle. Rova-T dose reductions were allowed. DLTs were evaluated in the DLT evaluation period, which occurred during the first three weeks of the first cycle of treatment. DLTs were defined as grade 4 thrombocytopenia (or grade 3 with bleeding) lasting >7 days or requiring platelet transfusion, grade 4 neutropenia lasting >7 days and/or requiring growth factor support, any febrile neutropenia, grade 4 anemia unrelated to underlying disease, clinically significant grade 3/4 nonhematologic laboratory abnormalities lasting >7 days, and grade 3/4 nonlaboratory AEs with the exception of fatigue, asthenia, nausea, or other constitutional symptoms. Grade ≥3 AEs clearly unrelated to study drug and grade ≥3 AEs of isolated alkaline phosphatase, amylase, or lipase laboratory abnormalities were not considered DLTs.

Disease assessments involved computed tomography (CT) scans of the chest, abdomen, pelvis, and neck (if indicated) and were conducted q6w during active study treatment for 24 weeks and every 12 weeks thereafter until disease progression. MRI scans of the brain were conducted if central nervous system progression was previously documented, and CT scans with intravenous contrast could be substituted at the discretion of the investigator. Patients with prostate cancer underwent whole-body technetium-99m bone scintigraphy. Tumor response was assessed by investigators according to RECIST v1.1, Response Assessment in Neuro-Oncology criteria for GBM, and Prostate Cancer Clinical Trials Working Group 3 (PCWG3) for prostate cancer^30^. AEs were summarized using preferred terms from the Medical Dictionary for Regulatory Activities and graded using the National Cancer Institute’s Common Terminology Criteria for Adverse Events version 4.03.

### Statistical analysis

The planned enrollment was ~144 patients in dose escalation and ~174 patients in dose expansion to detect an ORR of 15%, which would indicate efficacy worthy of further investigation. Efficacy was assessed by disease and cohort and included all patients who received at least one dose of Rova-T. BOR (defined as the best response of CR or PR, with confirmation not required), ORR (confirmed response), and DOR were summarized for all patients with a CR or PR according to RECIST v1.1 or PCWG3. DOR, PFS, and OS were evaluated using the Kaplan–Meier method. For DOR and PFS, patients were censored at the time at which they received another cancer therapy, missed two tumor assessments in a row, or had their last evaluable response assessment if not PD or death. Safety assessments were performed in all patients who received at least one dose of Rova-T.

### Reporting summary

Further information on research design is available in the Nature Research Reporting Summary linked to this article.

## Supplementary information

Supplementary Information

Reporting Summary

## Data Availability

AbbVie is committed to responsible data sharing regarding the clinical trials we sponsor. This includes access to anonymized, individual, and trial-level data (analysis data sets), as well as other information (e.g., protocols and Clinical Study Reports), as long as the trials are not part of an ongoing or planned regulatory submission. This includes requests for clinical trial data for unlicensed products and indications. This clinical trial data can be requested by any qualified researchers who engage in rigorous, independent scientific research and will be provided following review and approval of a research proposal and Statistical Analysis Plan (SAP) and execution of a Data Sharing Agreement (DSA). Data requests can be submitted at any time, and the data will be accessible for 12 months, with possible extensions considered. For more information on the process, or to submit a request, visit the following link: https://www.abbvie.com/our-science/clinical-trials/clinical-trials-data-and-information-sharing/data-and-information-sharing-with-qualified-researchers.html.
