# Supplementary material for: A phase I/II study of rovalpituzumab tesirine in delta-like 3—expressing advanced solid tumors
Source: NPJ Precis Oncol. 2021 Aug 5;5:74. doi: 10.1038/s41698-021-00214-y (PMC8342450; doi:10.1038/s41698-021-00214-y)
Supplement: Supplementary file 1 — Supplementary Information [file 41698_2021_214_MOESM1_ESM.pdf]

SUPPLEMENTARY INFORMATION

Supplementary Table 1. DLL3 Positivity Rate in Prescreening[1]

Cohort	Patients tested, n	No results, n	DLL3 positive (≥1%), n	DLL3 negative (<1%), n	DLL3 positivity rate, %
**GBM**	135	2	103	30	77
**GEP NEC**	161	15	100	46	68
**Large cell NEC**	87	3	63	21	75
**Melanoma**	150	19	65	66	50
**MTC**	35	2	33	0	100
**NEPC**	135	5	83	47	64
**Other NEC and NET**	243	14	136	93	59
**Other solid tumors**	347	23	158	166	49
**Total**	1293	83	741[2]	469	61

Abbreviations: GBM, glioblastoma; GEP NEC, gastroenteropancreatic neuroendocrine carcinoma; MTC, medullary thyroid carcinoma; NEC, neuroendocrine carcinoma; NEPC, neuroendocrine prostate cancer; NET, neuroendocrine tumor.

[1]Potential patients were pre-screened for DLL3 positivity to determine initial eligibility; those with DLL3-positive tumors underwent full screening for study eligibility upon disease progression.

[2]287 patients underwent full screening for study eligibility.

Supplementary Table 2. Primary diagnosis for other NEC/NET

**Primary diagnosis**
Neuroendocrine small cell carcinoma of the gallbladder
Other Neuroendocrine
Metastatic small cell neuroendocrine carcinoma and urothelial carcinoma of the anterior bladder wall
Invasive high-grade NEC of Bladder
Metastatic NEC of the gallbladder
Cervix
Small cell carcinoma
Carcinoid Lung
Supraglottic Larynx
Neuroendocrine small cell carcinoma of unconfirmed origin
Atypical thymic carcinoid tumor
Metastatic neuroendocrine carcinoma of colon primary
High grade neuroendocrine carcinoma of the lung
Merkel Cell Carcinoma
Small cell NEC of vagina
Carcinoma of the endocervix
Cervix
High grade neuroendocrine/small cell of mediastinum
Unknown primary neuroendocrine tumor with metastatic lesions to liver and bone
Lung adenocarcinoma
Lung
High Grade Neuroendocrine Carcinoma of Peritoneum
Thymic neuroendocrine
Lung
Small cell neuroendocrine carcinoma of the cervix
Metastatic neuroendocrine carcinoma of pulmonary primary
High-grade NEC tumor of the abdominal wall
Metastatic high-grade neuroendocrine carcinoma
Thymic
Small cell neuroendocrine carcinoma of the cervix
Neuroendocrine tumor of the lung

Abbreviations: NEC, neuroendocrine carcinoma; NET, neuroendocrine tumor.

Supplementary Table 3. Treatment-emergent SAEs in ≥2 patients treated at the 0.3-mg/kg dose

Preferred term, *n* (%)	Melanoma (*n* = 17)	MTC (*n* = 10)	GBM (*n* = 18)	Other solid tumor (*n* = 31)	Pooled NEC/NET (n = 69)	All patients treated at 0.3 mg/kg (*N* = 145)
**Any SAE**	8 (47)	3 (30)	8 (44)	19 (61)	39 (57)	77 (53)
**Malignant neoplasm progression**	1 (6)	0	3 (17)	2 (6)	12 (17)	18 (12)
**Pleural effusion**	2 (12)	0	0	2 (6)	3 (4)	7 (5)
**Pericardial effusion**	1 (6)	1 (10)	1 (6)	1 (3)	2 (3)	6 (4)
**Dyspnea**	1 (6)	1 (10)	0	3 (10)	0	5 (3)
**Dehydration**	0	0	0	4 (13)	0	4 (3)
**Fluid overload**	0	0	0	0	4 (6)	4 (3)
**Back pain**	0	0	0	1 (3)	3 (4)	4 (3)
**Acute kidney injury**	0	0	0	2 (6)	2 (3)	4 (3)
**Respiratory failure**	0	0	1 (6)	0	2 (3)	3 (2)
**Pulmonary embolism**	0	0	1 (6)	0	2 (3)	3 (2)
**Gastrointestinal hemorrhage**	0	0	0	2 (6)	1 (1)	3 (2)
**Nausea**	0	0	0	2 (6)	1 (1)	3 (2)
**Vomiting**	0	0	0	2 (6)	1 (1)	3 (2)
**Abdominal pain**	0	0	0	1 (3)	2 (3)	3 (2)
**Sepsis**	0	0	0	1 (3)	2 (3)	3 (2)
**Pyrexia**	0	0	0	0	3 (4)	3 (2)
**Acute respiratory failure**	1 (6)	0	0	0	1 (1)	2 (1)
**Pneumonia aspiration**	0	0	1 (6)	1 (3)	0	2 (1)
**Pneumonitis**	0	0	0	1 (3)	1 (1)	2 (1)
**Diarrhea**	0	0	0	1 (3)	1 (1)	2 (1)
**Hyperglycemia**	0	0	1 (6)	0	1 (1)	2 (1)
**Hyponatremia**	0	0	0	1 (3)	1 (1)	2 (1)
**Diverticulitis**	0	1 (10)	1 (6)	0	0	2 (1)
**Atrial fibrillation**	0	0	0	0	2 (3)	2 (1)
**Peripheral edema**	0	0	0	0	2 (3)	2 (1)
**Seizure**	0	0	2 (11)	0	0	2 (1)
**Confusional state**	0	0	0	1 (3)	1 (1)	2 (1)
**Mental status changes**	1 (6)	0	0	1 (3)	0	2 (1)

Abbreviations: GBM, glioblastoma; MTC, medullary thyroid carcinoma; NEC, neuroendocrine carcinoma; NET, neuroendocrine tumor; SAE, serious adverse event.

Supplementary Table 4. Adverse events of special interest in patients treated at the 0.3-mg/kg dose

	Melanoma (*n* = 17)		MTC (*n* = 10)		GBM (*n* = 18)		Other solid tumor (*n* = 31)		Pooled NEC/NET (*n* = 69)		All patients treated at 0.3 mg/kg (*N* = 145)	
**Preferred term, *n* (%)**	**All**	**Grade 3/4**	**All**	**Grade 3/4**	**All**	**Grade 3/4**	**All**	**Grade 3/4**	**All**	**Grade 3/4**	**All**	**Grade 3/4**
**Pleural effusion**	6 (35)	0	3 (30)	0	5 (28)	0	7 (23)	2 (6)	27 (39)	2 (3)	48 (33)	4 (3)
**Peripheral edema**	5 (29)	0	3 (30)	0	3 (17)	0	9 (29)	0	24 (35)	1 (1)	44 (30)	1 (1)
**Pericardial effusion**	5 (29)	0	4 (40)	1 (10)	5 (28)	1 (6)	5 (16)	1 (3)	19 (28)	2 (3)	38 (26)	5 (3)
**Photosensitivity reaction**	5 (29)	0	5 (50)	0	5 (28)	0	6 (19)	0	16 (23)	3 (4)	37 (26)	3 (2)
**Pneumonitis**	0	0	0	0	0	0	1 (3)	0	2 (3)	0	3 (2)	0

Abbreviations: GBM, glioblastoma; MTC, medullary thyroid carcinoma; NEC, neuroendocrine carcinoma; NET, neuroendocrine tumor.

Supplementary Table 5. TEAE leading to death in patients treated at the 0.3-mg/kg dose

Adverse event, *n* (%)	Melanoma (*n* = 17)	MTC (*n* = 10)	GBM (*n* = 18)	Other solid tumor (*n* = 31)	Pooled NEC/NET (*n* = 69)	All patients treated at 0.3 mg/kg (*N* = 145)
**TEAE leading to death, *n* (%)**	0	0	3 (17)	5 (16)	13 (19)	21 (14)
**Pneumonitis[1]**	0	0	0	1 (3)	1 (1)	2 (1)
**Multiple organ dysfunction**	0	0	0	1 (3)	0	1 (1)
**Acute respiratory failure[1]**	0	0	0	0	1 (1)	1 (1)
**Hepatic encephalopathy[1]**	0	0	0	1 (3)	0	1 (1)
**Device-related infection**	0	0	0	0	1 (1)	1 (1)
**Acute kidney injury**	0	0	0	1 (3)	0	1 (1)
**Malignant neoplasm progression**	0	0	3 (17)	2 (6)	9 (13)	14 (10)
**Neoplasm malignant**	0	0	0	0	1 (1)	1 (1)

Abbreviations: GBM, glioblastoma; MTC, medullary thyroid carcinoma; NEC, neuroendocrine carcinoma; NET, neuroendocrine tumor; TEAE, treatment-emergent adverse event.

[1]Considered drug related.

Supplementary Table 6. Efficacy in patients with NEC/NET at the 0.3-mg/kg dose

	LCNEC (*n* = 8)	NEPC (*n* = 14)	GEP NEC (*n* = 28)	Other NEC/NET[1] (*n* = 19)	Pooled NEC/NET (*N* = 69)
**ORR, *n* (%) (95% CI)**	0 (0.0–36.9)	1 (7) (0.2–33.9)	5 (18) (6.1–36.9)	3 (16) (3.4–39.6)	9 (13) (6.1–23.3)
**CR**	0	0	0	0	0
**PR**	0	1 (7)	5 (18)	3 (16)	9 (13)
**BOR, *n* (%)**	1 (13)	4 (29)	8 (29)	4 (21)	17 (25)
**CR**	0	0	0	0	0
**PR**	1 (13)	4 (29)	8 (29)	4 (21)	17 (25)
**Median DOR (95% CI), months[2]**	–	3 (2.8–3.1)	2.7 (1.2–NR)	–	3.1 (2.3–NR)
**Median PFS (95% CI), months**	4.4 (1.1–NR)	4.8 (2.7–5.7)	3.8 (2.3–5.0)	2.9 (1.3–NR)	4.1 (2.8–4.8)
**Median OS (95% CI), months**	4.9 (2.0–13.1)	6.4 (3.6–9.0)	8.1 (4.1–14.7)	10.8 (4.9–NR)	7.1 (5.6–9.7)

Abbreviations: BOR, best overall response; CR, complete response; DOR, duration of response; GEP NEC, gastroenteropancreatic neuroendocrine carcinoma; LCNEC, large-cell neuroendocrine carcinoma; NEC, neuroendocrine carcinoma; NET, neuroendocrine tumor; NEPC, neuroendocrine prostate cancer; NR, not reached; ORR, objective response rate; OS, overall survival; PFS, progression-free survival; PR, partial response.

[1]Patients with a BOR in the "Other NEC" category included 2 patients with lung carcinomas, 1 with metastatic small-cell NEC and urothelial carcinoma of the anterior bladder wall, and 1 with small-cell carcinoma; included in the ORR were 2 patients with lung carcinoma and 1 patient with metastatic small-cell NEC and urothelial carcinoma of the anterior bladder wall.

[2]DOR is defined as the time from first assessment on therapy of a CR or PR to the date of disease progression.

Supplementary Table 7. Efficacy at the 0.2-mg/kg dose

Outcome	Melanoma (*n* = 3)	MTC (*n* = 3)	GBM (*n* = 4)	Other solid tumor (*n* = 9)	Pooled NEC/NET (*n* = 24)
**ORR, *n* (%) (95% CI)**	1 (33.3) (0.8–90.6)	0 (0.0–70.8)	0 (0.0–60.2)	0 (0.0–33.6)	3 (12.5) (2.7–32.4)
CR	0	0	0	0	0
PR	1 (33.3)	0	0	0	3 (12.5)
**BOR, *n* (%)**	1 (33.3)	0	0	0	3 (12.5)
CR	0	0	0	0	0
PR	1 (33.3)	0	0	0	3 (12.5)
**Median DOR (95% CI), months[1]**	NR (NE–NE)	NA	NA	NA	2.6 (2.0–6.7)
**Median PFS (95% CI), months**	NR (1.2–NE)	NR (NE–NE)	1.9 (1.3–4.1)	1.4 (1.2–NE)	4.3 (1.3–5.5)
**Median OS (95% CI), months**	7.9 (3.3–9.2)	NR (NE–NE)	5.4 (1.9–6.7)	13.4 (1.2–NE)	5.0 (2.6–6.0)

Abbreviations: BOR, best overall response; CI, confidence interval; CR, complete response; DOR, duration of response; GBM, glioblastoma; MTC, medullary thyroid carcinoma; NA, not assessable; NE, not estimable; NEC, neuroendocrine carcinoma; NET, neuroendocrine tumor; NR, not reached; ORR, objective response rate; OS, overall survival; PFS, progression-free survival; PR, partial response.

[1]DOR is defined as the time from first assessment on therapy of a CR or PR to the date of disease progression.

Supplementary Table 8. Efficacy at the 0.4-mg/kg dose

Outcome	Melanoma (*n* = 0)	MTC (*n* = 0)	GBM (*n* = 1)	Other solid tumor (*n* = 3)	Pooled NEC/NET (*n* = 8)
**ORR, *n* (%) (95% CI)**	0	0	0 (0.0–97.5)	0 (0.0–70.8)	2 (25) (3.2–65.1)
CR	0	0	0	0	0
PR	0	0	0	0	2 (25)
**BOR, *n* (%)**	0	0	0	0	3 (37.5)
CR	0	0	0	0	0
PR	0	0	0	0	3 (37.5)
**Median DOR (95% CI), months[1]**	NA	NA	NA	NA	11.4 (0.5–NE)
**Median PFS (95% CI), months**	NA	NA	NR (NE–NE)	2.8 (2.1–3.4)	3.0 (0.6–NE)
**Median OS (95% CI), months**	NA	NA	NR (NE–NE)	5.9 (2.1–5.9)	3.0 (0.6–NE)

Abbreviations: BOR, best overall response; CI, confidence interval; CR, complete response; DOR, duration of response; GBM, glioblastoma; MTC, medullary thyroid carcinoma; NA, not assessable; NE, not estimable; NEC, neuroendocrine carcinoma; NET, neuroendocrine tumor; NR, not reached; ORR, objective response rate; OS, overall survival; PFS, progression-free survival; PR, partial response.

[1]DOR is defined as the time from first assessment on therapy of a CR or PR to the date of disease progression.

Supplementary Figure 1. Best Change in Tumor Lesion Size for Melanoma (n = 18)[1]

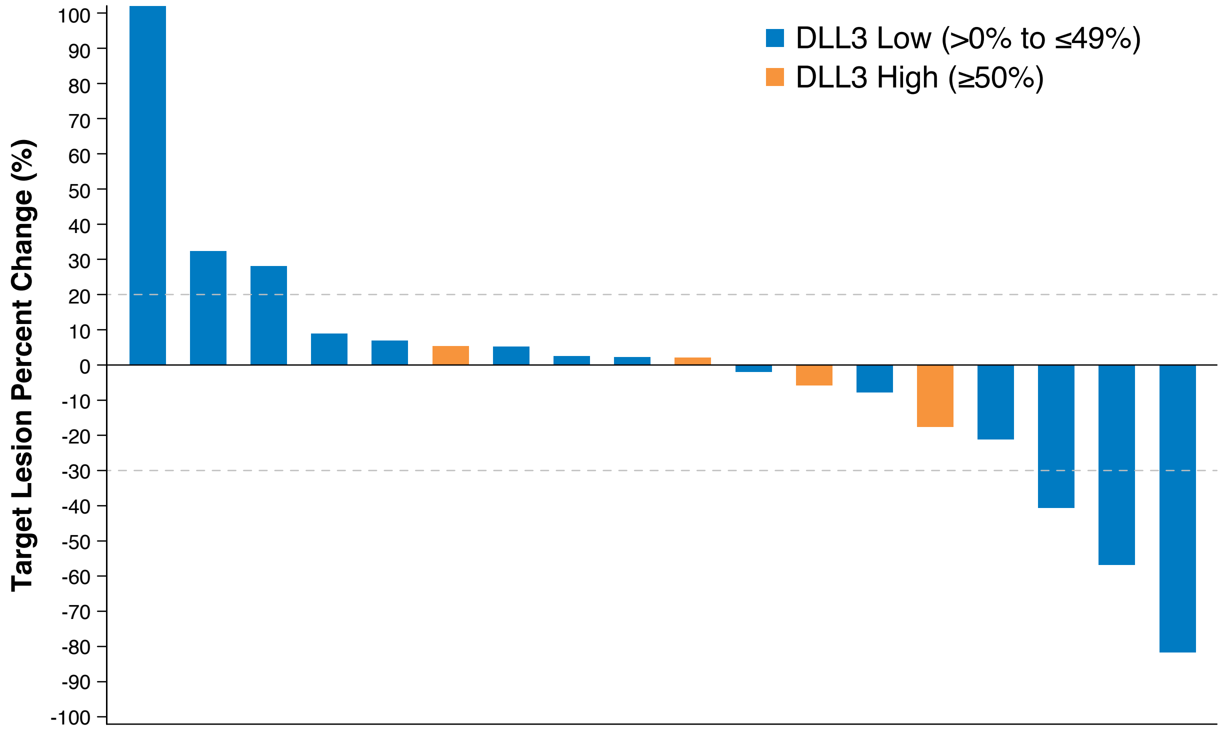

[1]All patients with melanoma treated at any dose level.
Abbreviations: DLL3, delta-like protein 3.

Supplementary Figure 2. Best Change in Tumor Lesion Size for MTC (n = 12)[1]

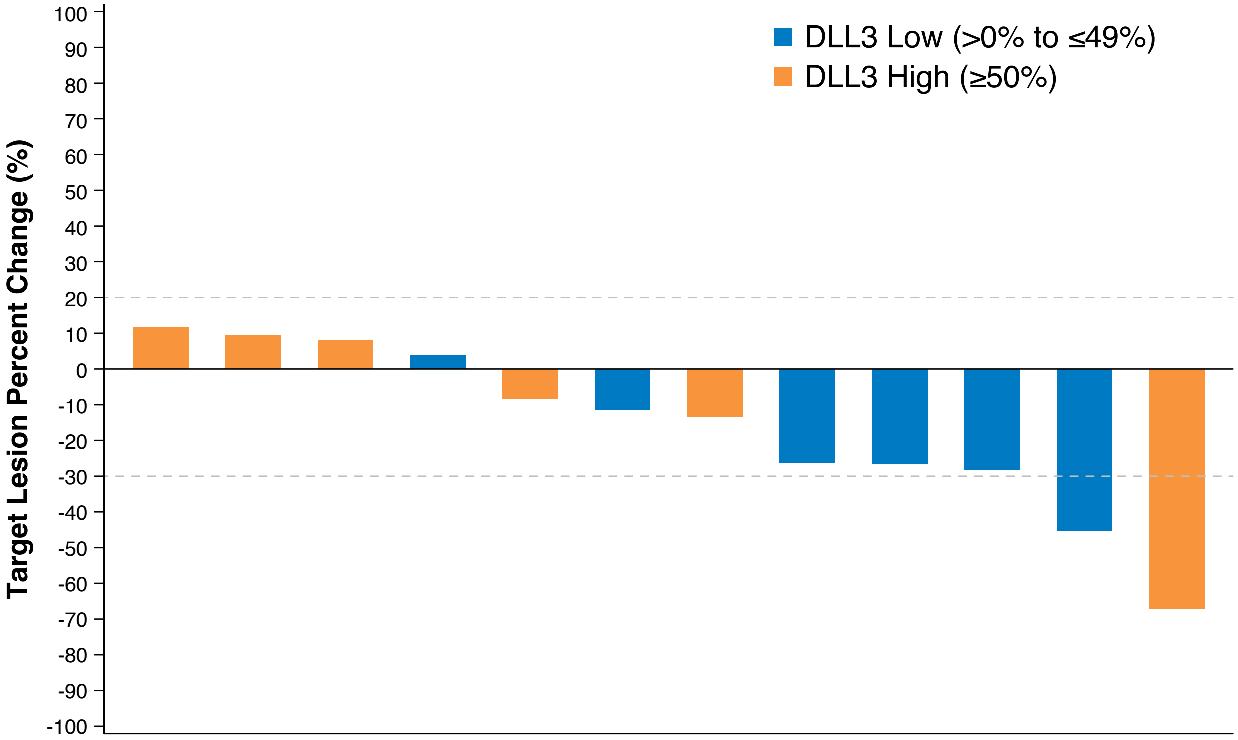

[1]All patients with MTC treated at any dose level.
Abbreviations: DLL3, delta-like protein 3; MTC, medullary thyroid carcinoma.

Supplementary Figure 3. Best Change in Tumor Lesion Size for GBM (n = 16)[1]

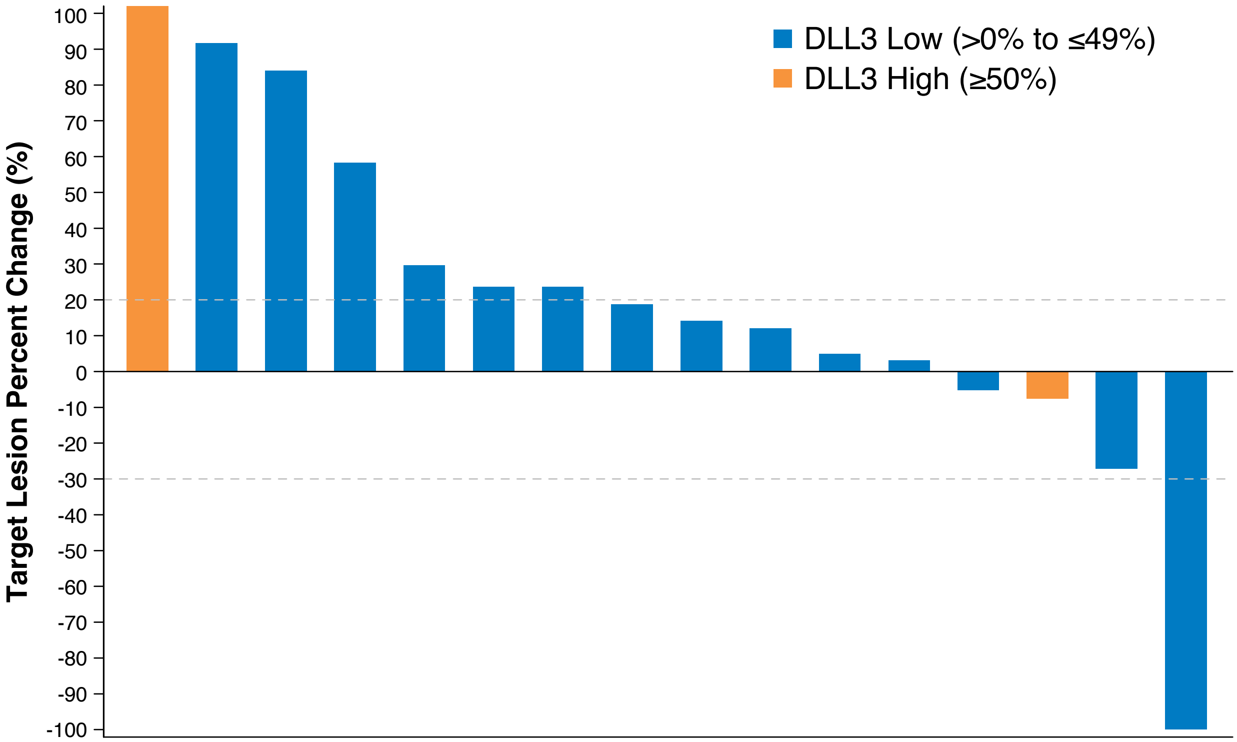

[1]All patients with GBM treated at any dose level.
Abbreviations: DLL3, delta-like protein 3; GBM, glioblastoma.

Supplementary Figure 4. Best Change in Tumor Lesion Size for Other Solid Tumor (n = 34)[1]

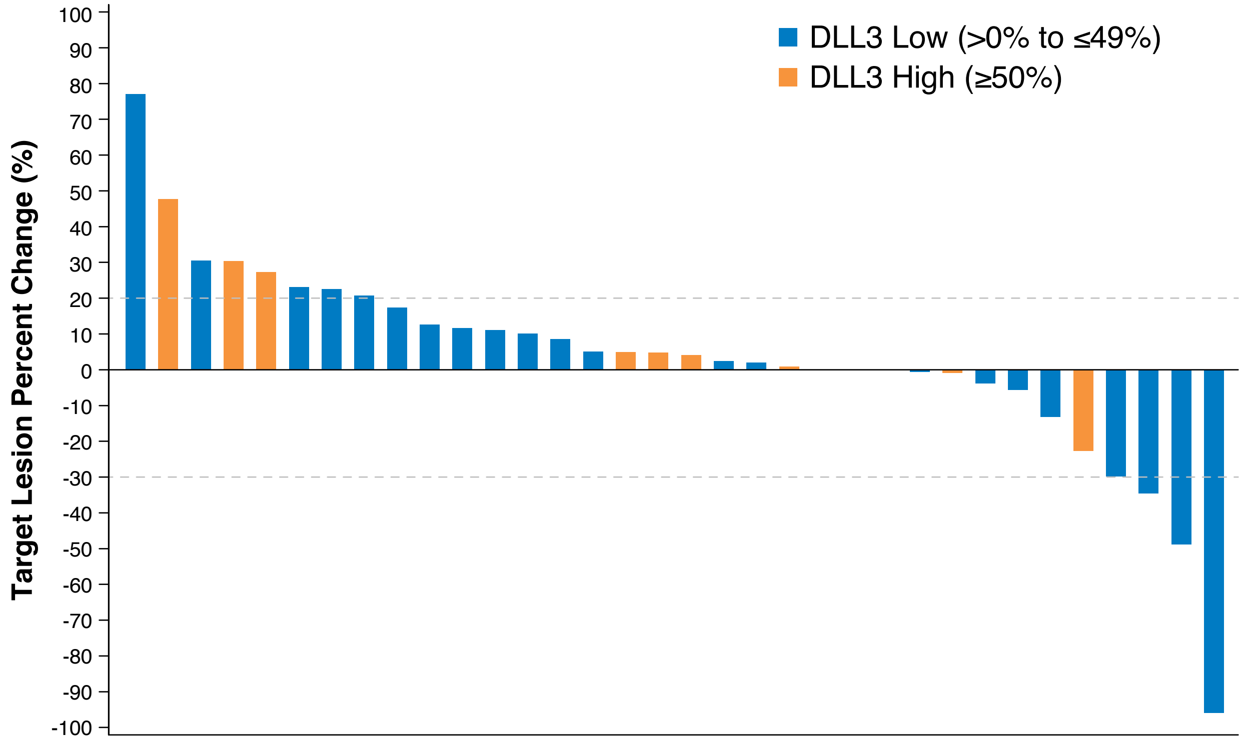

[1]All patients with other solid tumors treated at any dose level.
Abbreviations: DLL3, delta-like protein 3.

Supplementary Figure 5. Best Change in Tumor Lesion Size for LCNEC (n = 12)[1]

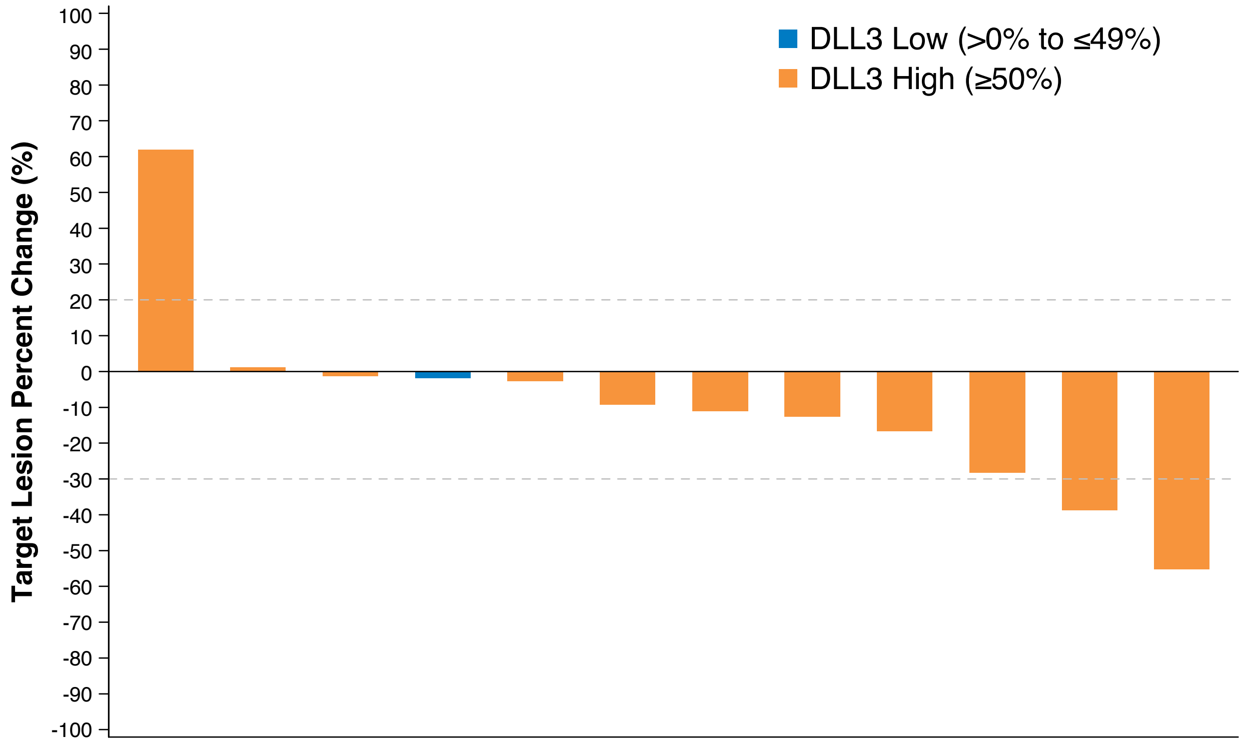

[1]All patients with large cell NEC treated at any dose level.
Abbreviations: DLL3, delta-like protein 3; LCNEC, large-cell neuroendocrine carcinoma.

Supplementary Figure 6. Best Change in Tumor Lesion Size for NEPC (n = 18)[1]

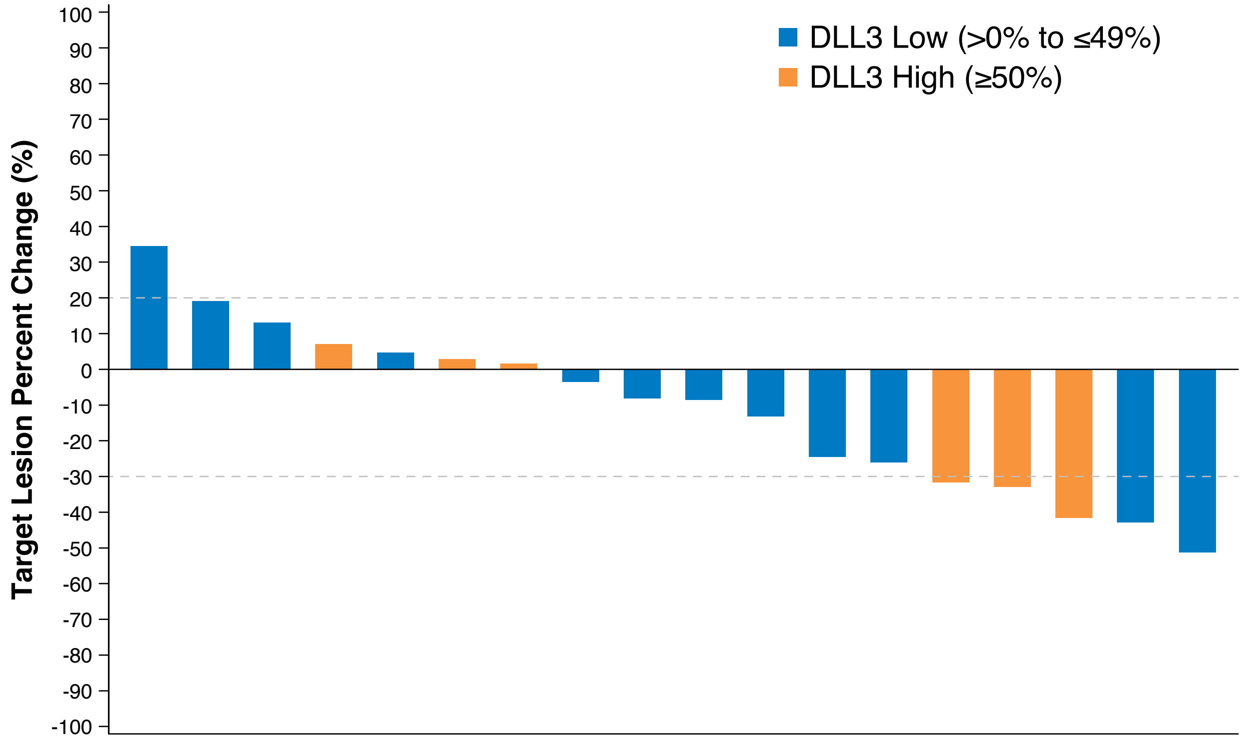

[1]All patients with neuroendocrine prostate cancer treated at any dose level.
Abbreviations: DLL3, delta-like protein 3; NEPC, neuroendocrine prostate cancer.

Supplementary Figure 7. Best Change in Tumor Lesion Size for GEP NEC (n = 25)[1]

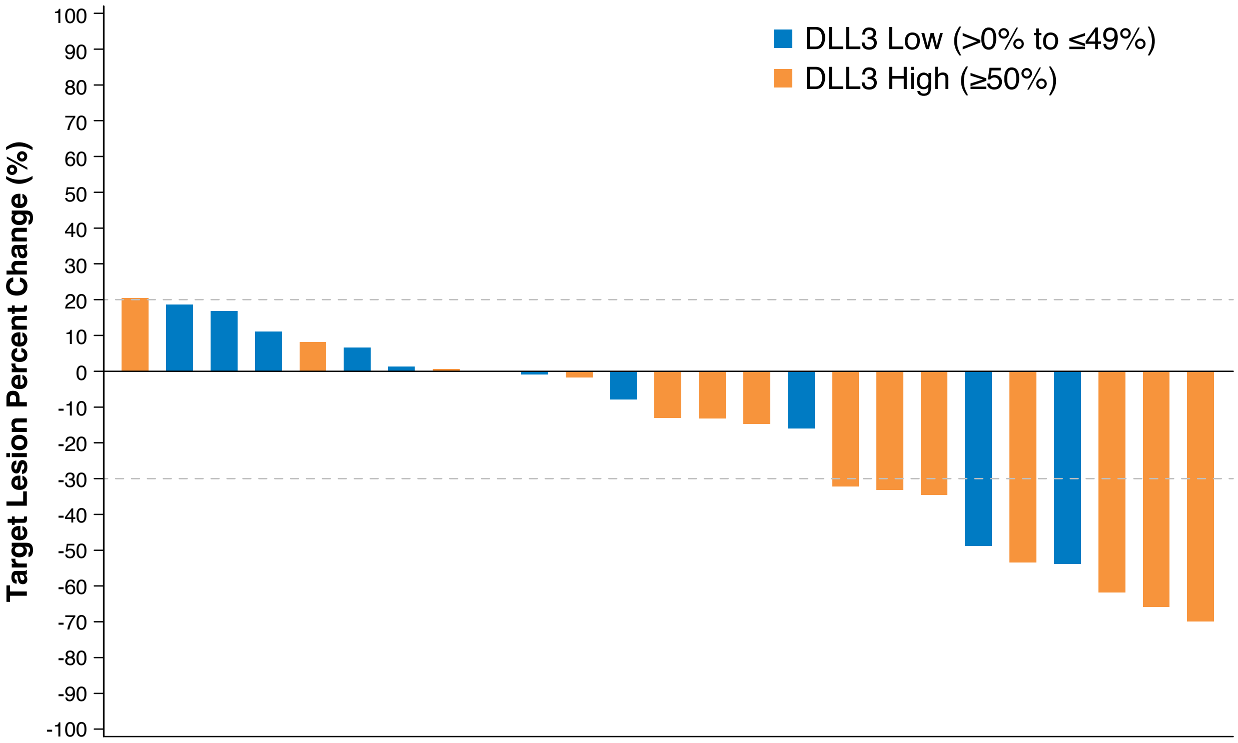

[1]All patients with GEP NEC treated at any dose level.
Abbreviations: DLL3, delta-like protein 3; GEP NEC, gastroenteropancreatic neuroendocrine carcinoma.

Supplementary Figure 8. Best Change in Tumor Lesion Size for Other NEC/high-grade NET (n = 28)[1]

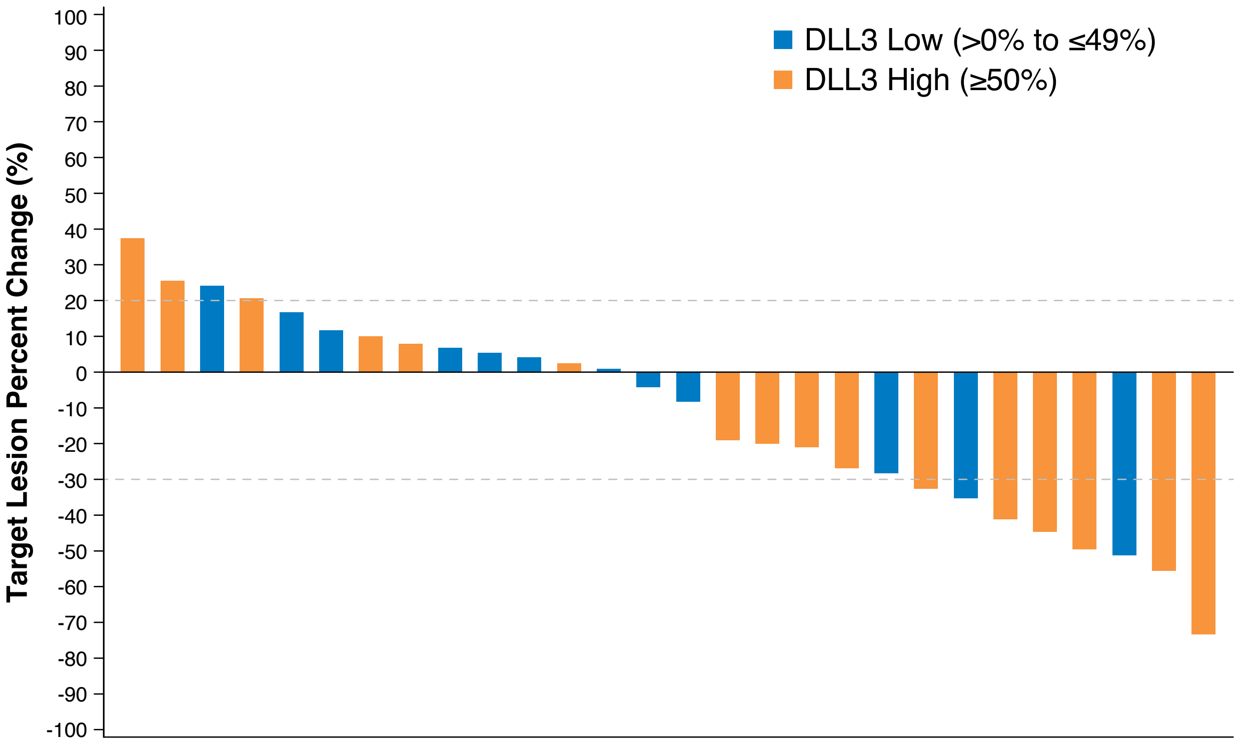

[1]All patients with other NEC/high-grade NET treated at any dose level.
Abbreviations: DLL3, delta-like protein 3; NEC, neuroendocrine carcinoma; NET, neuroendocrine tumor.
