# Supplementary material for: A phase I/II study of rovalpituzumab tesirine in delta-like 3—expressing advanced solid tumors
Source: NPJ Precis Oncol. 2021 Aug 5;5:74. doi: 10.1038/s41698-021-00214-y (PMC8342450; doi:10.1038/s41698-021-00214-y)
Supplement: Supplementary file 2 — Reporting Summary [file 41698_2021_214_MOESM2_ESM.pdf]

nature research

Corresponding author(s): Rahul Aggarwal
UCSF Helen Diller Family Comprehensive Cancer Center
Rahul.Aggarwal@ucsf.edu

Last updated by author(s): June 28, 2021

Reporting Summary

Nature Research wishes to improve the reproducibility of the work that we publish. This form provides structure for consistency and transparency in reporting. For further information on Nature Research policies, see our Editorial Policies and the Editorial Policy Checklist.

Statistics

For all statistical analyses, confirm that the following items are present in the figure legend, table legend, main text, or Methods section.

n/a	Confirmed	
☐	☒	The exact sample size (*n*) for each experimental group/condition, given as a discrete number and unit of measurement
☒	☐	A statement on whether measurements were taken from distinct samples or whether the same sample was measured repeatedly
☒	☐	The statistical test(s) used AND whether they are one- or two-sided<br>*Only common tests should be described solely by name; describe more complex techniques in the Methods section.*
☐	☒	A description of all covariates tested
☒	☐	A description of any assumptions or corrections, such as tests of normality and adjustment for multiple comparisons
☐	☒	A full description of the statistical parameters including central tendency (e.g. means) or other basic estimates (e.g. regression coefficient) AND variation (e.g. standard deviation) or associated estimates of uncertainty (e.g. confidence intervals)
☒	☐	For null hypothesis testing, the test statistic (e.g. *F*, *t*, *r*) with confidence intervals, effect sizes, degrees of freedom and *P* value noted<br>*Give P values as exact values whenever suitable.*
☒	☐	For Bayesian analysis, information on the choice of priors and Markov chain Monte Carlo settings
☒	☐	For hierarchical and complex designs, identification of the appropriate level for tests and full reporting of outcomes
☒	☐	Estimates of effect sizes (e.g. Cohen's *d*, Pearson's *r*), indicating how they were calculated

Our web collection on statistics for biologists contains articles on many of the points above.

Software and code

Policy information about availability of computer code

Data collection	N/A
Data analysis	N/A

For manuscripts utilizing custom algorithms or software that are central to the research but not yet described in published literature, software must be made available to editors and reviewers. We strongly encourage code deposition in a community repository (e.g. GitHub). See the Nature Research guidelines for submitting code & software for further information.

Data

Policy information about availability of data

All manuscripts must include a data availability statement. This statement should provide the following information, where applicable:

- Accession codes, unique identifiers, or web links for publicly available datasets
- A list of figures that have associated raw data
- A description of any restrictions on data availability

AbbVie is committed to responsible data sharing regarding the clinical trials we sponsor. This includes acces to anonymized, individual and trial-level data (analysis data sets), as well as other information (eg, protocols and Clinical Study Reports), as long as the trials are not part of an ongoing or planned regulatory submission. This includes requests for clinical trial data for unlicensed products and indications.
This clinical trial data can be requested by any qualified researchers who engage in rigorous, independent scientific research and will be provided following review and approval of a research proposal and Statistical Analysis Plan (SAP) and execution of a Data Sharing Agreement (DSA). Data requests can be submitted at any time, and the data will be accessible for 12 months, with possible extensions considered. For more information on the process, or to submit a request, visit the following link: https://www.abbvie.com/our-science/clinical-trials/clinical-trials-data-and-information-sharing/data-and-information-sharing-with-qualified-researchers.html.

Field-specific reporting

Please select the one below that is the best fit for your research. If you are not sure, read the appropriate sections before making your selection.

☒ Life sciences ☐ Behavioural & social sciences ☐ Ecological, evolutionary & environmental sciences

For a reference copy of the document with all sections, see nature.com/documents/nr-reporting-summary-flat.pdf

Life sciences study design

All studies must disclose on these points even when the disclosure is negative.

Sample size	The planned enrollment was approximately 144 patients in dose escalation and approximately 174 patients in dose expansion to detect an ORR of 15%, which would indicate efficacy worthy of further investigation.
Data exclusions	For DOR and PFS, patients were censored at the time at which they received another cancer therapy, missed two tumor assessments in a row, or had their last evaluable response assessment if not PD or death.
Replication	These findings were not replicated because the Rova-T program was terminated.
Randomization	This study was not randomized
Blinding	This study was not blinded

Reporting for specific materials, systems and methods

We require information from authors about some types of materials, experimental systems and methods used in many studies. Here, indicate whether each material, system or method listed is relevant to your study. If you are not sure if a list item applies to your research, read the appropriate section before selecting a response.

Materials & experimental systems

n/a	Involved in the study	
☐	☒	Antibodies
☒	☐	Eukaryotic cell lines
☒	☐	Palaeontology and archaeology
☒	☐	Animals and other organisms
☐	☒	Human research participants
☐	☒	Clinical data
☒	☐	Dual use research of concern

Methods

n/a	Involved in the study	
☒	☐	ChIP-seq
☒	☐	Flow cytometry
☒	☐	MRI-based neuroimaging

Antibodies

Antibodies used	SC16.65 mouse antibody
Validation	The delta-like protein 3 (DLL3) murine monoclonal antibody, SC16.65, was evaluated on the Ventana platform to confirm specificity and sensitivity with immunohistochemistry (IHC) in formalin fixed paraffin embedded (FFPE) samples. SC16.65 specifically recognized DLL3 in DLL3 overexpressing 293T cells but was negative when evaluated on naïve 293T cells which demonstrated specific immunoreactivity of SC16.65 to overexpressed DLL3. Specificity was further evaluated and confirmed using CRISPR DLL3 knockout COLO679 clonal cell lines which were negative by SC16.65 IHC whereas parental COLO679 cells demonstrated strong and specific DLL3 immunoreactivity. The dynamic range of DLL3 expression with SC16.65 IHC was determined with DLL3 endogenously expressing cell lines that are positive by SC16.65 IHC down to CCLE mRNA level of 6.5 and Western blot protein level of 48 by densitometry, a ratio of 0.21 relative to the highest expresser SHP77. Incremental expression of DLL3 in high, medium, and low DLL3 endogenously expressing cell lines by SC16.65 IHC demonstrated a correlation with orthogonal data. Human tissue was evaluated with the SC16.65 IHC assay and demonstrated that normal lung was negative for DLL3 whereas SCLC patient samples had a range of DLL3 expression (0-100%; median=30) with 88.5% of patient samples expressing DLL3 ≥1% of neoplastic cells, and 39.4% ≥50% DLL3 high expression.

Human research participants

Policy information about studies involving human research participants

Population characteristics	There were 101 patients with high-grade NETs/NECs (pulmonary and extrapulmonary large cell NEC [n = 13], NEPC [n = 21], high-grade GEP NETs [n = 36], and other NECs [n = 31]) and 99 patients with other solid tumors (melanoma [n = 20], MTC [n = 13], GBM [n = 23], and other [n = 43]). The median age was 61 (range, 28–84) years, and 94% of patients had stage IV disease at study entry. Seventy-seven (39%) patients had tumors expressing a high level of DLL3, which was defined as [illegible]50% DLL3-

	positive cells. Most patients (55%) had received three or more prior therapies. The median duration of follow-up was 4.6 (range, 0.1–33.7) months in all patients and 4.7 (range 0.1 – 27.1) months in patients treated at the RP2D of 0.3mg/kg.
Recruitment	Patients were recruited by participating institutions if they met pre-specified eligibility criteria. Adult patients with unresectable, refractory, advanced solid tumors other than SCLC who were positive for DLL3 and had measurable disease were included in the study. DLL3 positivity was defined as immunohistochemical staining in ≥1% of tumor cells. Potential patients were pre-screened for DLL3 positivity to determine initial eligibility, and those with DLL3-positive tumros underwent full screening for study eligibility upon disease progresion. Measurable disease was defined based on Response Evaluation Criteria in Solid Tumors version 1.1 (RECIST v1.1)30. Patients had to have an Eastern Cooperative Oncology Group (ECOG) performance status of 0 to 1, a life expectancy of ≥12 weeks, and satisfactory laboratory parameters. Patients could not have a clinically significant medical condition, including uncontrolled hypertension and/or diabetes, pulmonary disease, neurological disorder, recent or ongoing serious infection, or a cerebral vascular event within 6 months of starting the study. Prior exposure to pyrrolobenzodiazepine-containing drugs, including Rova-T, was not allowed. All patients provided written informed consent.
Ethics oversight	The study was conducted according to the Declaration of Helsinki and all applicable laws, rules, and regulations within the relevant jurisdictions of the investigators; the study was approved by the institutional review boards at each participating institution.

Note that full information on the approval of the study protocol must also be provided in the manuscript.

Clinical data

Policy information about clinical studies

All manuscripts should comply with the ICMJE guidelines for publication of clinical research and a completed CONSORT checklist must be included with all submissions.

Clinical trial registration	NCT02709889
Study protocol	The protocol is provided as an addition to the supplementary materials for publication along with the manuscript.
Data collection	Patients were enrolled between September 2016 and February 2019
Outcomes	The primary endpoint was safety. Secondary endpoints included BOR, ORR, duration of response (DOR), PFS, and OS. The relationship between DLL3 expression and clinical outcome was tested as an exploratory endpoint. AEs were summarized using preferred terms from the Medical Dictionary for Regulatory Activities and graded using the National Cancer Institute's Common Terminology Criteria for Adverse Events version 4.03. Disease assessments involved computed tomography (CT) scans of the chest, abdomen, pelvis, and neck (if indicated) and were conducted q6w during active study treatment for 24 weeks and every 12 weeks thereafter until disease progression. MRI scans of the brain were conducted if central nervous system progression was previously documented, and CT scans with intravenous contrast could be substituted at the discretion of the investigator. Patients with prostate cancer underwent whole-body technetium-99m bone scintigraphy. Tumor response was assessed by investigators according to RECIST v1.1, Response Assessment in Neuro-Oncology criteria for GBM, and Prostate Cancer Clinical Trials Working Group 3 (PCWG3) for prostate cancer.
